# Assessing the Risk of Respiratory-Related Healthcare Visits Associated with Wildfire Smoke Exposure in Children 0–18 Years Old: A Systematic Review

**DOI:** 10.3390/ijerph18168799

**Published:** 2021-08-20

**Authors:** Shelby Henry, Maria B. Ospina, Liz Dennett, Anne Hicks

**Affiliations:** 1 Faculty of Medicine and Dentistry, University of Alberta, Edmonton, AB T6G 2R3, Canada; slhenry@ualberta.ca; 2 Department of Obstetrics and Gynecology, University of Alberta, 220 B Heritage Medical Research Centre, Edmonton, AB T6G 2S2, Canada; mospina@ualberta.ca; 3 Scott Health Sciences Library, University of Alberta, Edmonton, AB T6G 2R7, Canada; liz.dennett@ualberta.ca; 4 Department of Pediatrics, University of Alberta, 4-590 ECHA 11405 87 Ave, Edmonton, AB T6G 1C9, Canada

**Keywords:** wildfire, pediatric, respiratory disease

## Abstract

Wildfires are increasing in frequency, size, and intensity, and increasingly affect highly populated areas. Wildfire smoke impacts cardiorespiratory health; children are at increased risk due to smaller airways, a higher metabolic rate and ongoing development. The objective of this systematic review was to describe the risk of pediatric respiratory symptoms and healthcare visits following exposure to wildfire smoke. Medical and scientific databases and the grey literature were searched from inception until December 2020. Included studies evaluated pediatric respiratory-related healthcare visits or symptoms associated with wildfire smoke exposure. Prescribed burns, non-respiratory symptoms and non-pediatric studies were excluded. Risk of bias was evaluated using the National Toxicology Program’s Office of Health Assessment and Translation Risk of Bias Rating Tool. Data are presented narratively due to study heterogeneity. Of 2138 results, 1167 titles and abstracts were screened after duplicate removal; 65 full text screens identified 5 pre-post and 11 cross-sectional studies of rural, urban and mixed sites from the USA, Australia, Canada and Spain. There is a significant increase in respiratory emergency department visits and asthma hospitalizations within the first 3 days of exposure to wildfire smoke, particularly in children < 5 years old.

## 1. Introduction

Climate change influences extreme weather events, contributing to global natural disasters, including wildfires. Heatwaves, changes to precipitation leading to increased incidence of flooding and drought, as well as increased intensity of windstorms all increase the risk of uncontrolled fires [1]. Historically, the driving force of wildfires was precipitation level; however, modelling predicts that a shift to temperature-driven wildfires has begun to occur and will continue throughout the 21st century [2]. Wildfires have been increasing in frequency, size and intensity [3], with the number of unmanageable crown fires projected to continue increasing throughout the remainder of the 21st century [4,5,6]. The wildfire burning season is also expected to increase, with more days of uncontrolled burning in which the intensity exceeds the ability to suppress the fire [4]. Nearly 4000 wildfires burned in Canada in 2019, with the area burned exceeding 1.8 million hectares [6].

Wildfires negatively impact the environment, climate, economy and importantly, human health. Smoke produced by wildfires is composed of small particulate matter and toxic gases that are harmful to human health when inhaled. Composition of the smoke is dependent on many factors including: temperature of the fire, how long it burns, the fuel source/vegetation burned, the weather, and how far the smoke has travelled from the fire source [7]. In any type of air pollution, of most concern is the small particulate matter (PM_2.5_) that can be inhaled into bronchioles and alveoli, where it causes local irritation and damage [8,9]. Systemic impacts are also observed, including effects on pregnancy outcomes such as preterm birth [10]. Wildfire smoke significantly increases PM_2.5_ levels in the air, spreading long distances from the source and remaining elevated for weeks [11,12]. Regardless of the source, increased levels of PM_2.5_ in the air have adverse health effects such as increased cardiorespiratory morbidity and mortality and public health burdens [13,14], including cost and increased numbers of healthcare visits. This adds a significant financial burden to the healthcare system from potentially preventable use of resources.

Previous systematic reviews that included participants of all ages found that exposure to wildfire smoke significantly increased respiratory morbidity. A small number of studies have investigated the risk of respiratory-related healthcare utilization specifically in children [15,16,17]. It has been suggested that children may be at an increased risk of negative respiratory effects from wildfire smoke due to their smaller airway size and developing lungs [18,19]. Additionally, parents of young children may be more likely to access healthcare for their child’s respiratory symptoms compared to the average adult. The primary objective of this systematic review was to synthesize the data from studies investigating the risk of respiratory-related healthcare visits specifically among children aged 0–18 years old following exposure to wildfire smoke. The secondary objective was to pool data from primary studies reporting respiratory (both upper and lower respiratory tract) symptoms in children following exposure to wildfire smoke. We hypothesized that respiratory-related symptoms and healthcare visits will increase significantly in children following wildfire smoke exposure, and that younger children (<5) will demonstrate increased risk of healthcare visits compared to older children and teenagers.

## 2. Materials and Methods

### 2.1. Protocol, Registration and Search Strategy

This systematic review follows the Preferred Reporting Items for Systematic Reviews and Meta-Analyses (PRISMA) guidelines [20]; Appendix A Table A1. The study protocol was registered with PROSPERO (CRD: 188705). A health sciences librarian (LD) conducted comprehensive searches of biomedical databases from database inception to December 2020: Medline (Ovid MEDLINE(R) ALL), Embase (Ovid interface), CINAHL Plus with Full Text (EBSCOhost interface), Greenfile (EBSCOhost interface), Web of Science (Indexes = SCI-EXPANDED, SSCI, A&HCI, CPCI-S, CPCI-SSH, BKCI-S, BKCI-SSH, ESCI, CCR-EXPANDED, IC), CABI: CAB Abstracts and Global Health (Clarivate Analytics interface), Proquest Earth, Atmospheric & Aquatic Science Database, Scopus, and HERO- Health and Environmental Research Online from database inception until 21 December 2020. The search combined a list of keyword synonyms for wildfires with a modified search hedge for pediatric studies [21]. No date, language, or study design limits were used. Google and Google Scholar searches and contacts with experts in the field were conducted and reference lists of reviews and included articles were reviewed to identify additional studies. The full details of the search strategy can be found in Supplement S1.

### 2.2. Eligibility Criteria and Study Selection

Peer-reviewed primary research on wildfires and pediatric respiratory health published up to 21 December 2020 was reviewed; the inclusion criteria are described using the Population, Exposure, Comparison, Outcome, Study Design (PECOS) framework for environmental health reviews [22]. To be included, studies must have included and independently described a population of children between 0–20 years old (increased from 0–18 years old described in the PROSPERO protocol to include additional studies). Exposures were characterized as specifically exposure to smoke produced by wildfires burning any vegetation. Measures of exposure were direct, through air sampling devices deployed for the study or standardized reporting from existing local air quality monitors, or indirect through satellite imagery, visibility index or self-reported perception of exposure, and the exposed population was designated by postal or zip code, county, or address of residence. Outcomes included respiratory-related ambulatory, Emergency Department (ED) and hospitalization-related healthcare visits and/or respiratory symptoms. Comparison populations included: similar populations during the same time period that were not exposed, the same population at a different time point when the exposure was not present, or healthcare visits in the exposed population during the exposure time that were not attributable to wildfire exposure (e.g., fractures). Studies regarding prescribed burning, indoor/outdoor controlled wood burning, or wildfires resulting from a separate primary disaster (e.g., volcanic eruption) were excluded. As this review intended to focus on wildfire smoke and healthcare utilization, studies that focused solely on non-smoke-related outcomes (e.g., burns) and mortality/fatality were excluded. Studies that exclusively reported pregnancy and birth-related outcomes were also excluded. Included study designs were observational studies (prospective and retrospective cohort, cross-sectional, case–control, ecological and time series). Case-reports and case-series, reviews, simulation studies, letters to the editor and commentaries were excluded. Studies with a high risk of bias in all domains of the PECOS question were excluded. We included all papers that had full text available in English or translatable by Google Translate, as translation services were not feasible for this review. For duplicate or overlapping studies the most recent article was used and the rest excluded. Full texts of relevant articles were retrieved and screened independently by the same reviewers for inclusion; disagreements regarding study inclusion were resolved by discussion between reviewers (SH and AH) until consensus was reached [20]. All studies underwent title and abstract screening for relevance, followed by a full-text review and risk of bias assessment conducted in duplicate by two independent reviewers (SH, AH). Titles with inconclusive title/abstract results or disagreements were retrieved for full text review. Disagreements about full-text study inclusion were resolved by consensus [20]. Complete references of excluded studies and reasons for exclusion are available on request.

### 2.3. Data Collection

Data were extracted independently by one reviewer (SH) with a second reviewer (AH) verifying accuracy. Study authors were contacted for additional information as required. Information regarding study characteristics (i.e., study date, duration, design, location), population characteristics (sample size, sex, age at assessment), exposure characteristics (exposure measures including number of smoke days, PM_2.5_, PM_10_, ozone, visibility and perceived smoke exposure and measurement strategies including type and location of ground-based sampling devices, satellite imagery and data sources) and outcome assessment (symptoms, outpatient clinic, ED visits or hospitalizations for respiratory presentations) were extracted from individual studies using a data collection form designed prior to the literature search. Quantitative data were extracted when studies reported outcomes as odds ratio (OR), risk ratio (RR), or the number of excess health care visits attributable to the exposure. For one study, the original data were not available [23]; the principal author recommended measuring the graph to estimate OR and confidence intervals. The published graph was magnified to 400% and measured; estimated results were obtained by comparing data points to the y axis. Results were described using mean, standard deviation for continuous data and proportions and percentages for categorical data. Google Sheets was used to track data. Meta-analysis was not conducted due to significant heterogeneity in study design, exposure and outcome evaluation and reporting between included studies [24].

### 2.4. Risk of Bias, Evidence Synthesis and Certainty

Risk of bias of individual studies was evaluated by two independent reviewers (SH and AH) using the National Toxicology Program’s Office of Health Assessment and Translation (OHAT) Risk of Bias Rating Tool for Human and Animal Studies [25,26,27]. The OHAT tool evaluates six domains for bias at the outcome level using 11 questions that address selection, confounding, attrition/exclusion, performance, detection and selective reporting bias, without excluding low quality studies [27]. For this review of observational studies, participant selection, confounding, exposure measurement, outcome assessment, follow up and completeness of outcome reporting were assessed as: probably or possibly low, possibly high or probably high risk of bias based on consensus; discrepancies were resolved through discussion.

The Systematic Review Without Meta-analysis (SWIM) guidelines were used to report data and for evidence synthesis (Table A2). Study characteristics and risk of bias were described narratively and summarized in tables and figures. An effect direction plot was developed to present an overview of the information, using a vote-counting approach supported by the tabular data to summarize the direction of identified associations [10]. All reported results were considered to have no significant association unless the confidence interval did not cross an OR or RR of 1.0; significant numbers below 1.0 were reported as a negative association and above 1.0 a positive association. We used the adapted Grading of Recommendations Assessment, Development and Evaluation (GRADE) framework for environmental health reviews [28] to assess the certainty of the evidence as high, moderate, low, or very low. Parameters that increased certainty were evidence of a dose–response relationship and larger effect size; higher risk of bias, small sample or effect sizes, wide confidence intervals and poor relevance of the study to the PECO questions were criteria used to downgrade the certainty of evidence. Disagreements were resolved by consensus.

## 3. Results

### 3.1. Search Results

**Figure 1:**
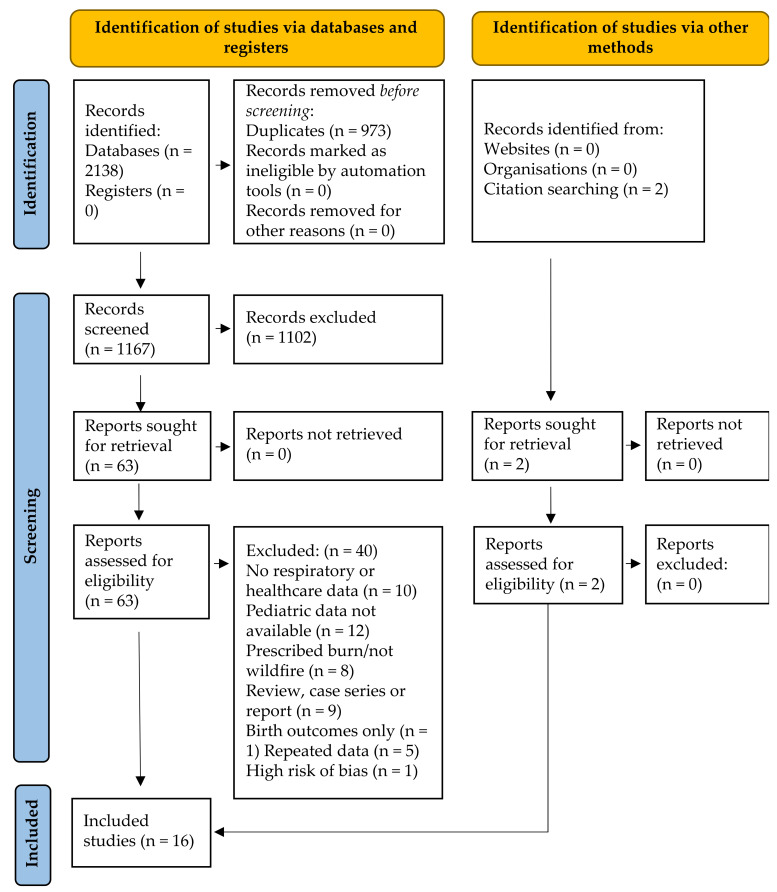
Modified PRISMA flow diagram [20] for pediatric outcomes associated with wildfire smoke exposure resulting from searches of databases, registers and other sources.

The electronic and grey literature search identified 2138 studies; 1165 after duplicate removal. No grey literature studies were identified. The detailed study selection process is outlined in Figure 1. After title and abstract screening, 1102 of the 1165 identified studies were excluded. No grey literature studies were identified. Of the 65 studies included in full-text screening, 10 were excluded due to no respiratory or healthcare outcomes, 12 due to no pediatric (or undifferentiated) data, 8 due to prescribed burn or other non-wildfire exposure, 9 due to study methodology (case-series, case report or review), 3 because no full text was available, 1 only assessed birth outcomes and 5 repeated data presented in included studies. One of the 17 remaining studies was excluded for high risk of bias [29] in all domains. The 16 studies included in this review are summarized in Table 1.

### 3.2. Study and Population Characteristics

Included studies encompassing data collected between 1996 and 2017 were published between 2006 and 2020. Four pre/post [30,35,38,39], three cross-sectional case crossover [15,34,40] and nine cross-sectional [16,23,31,33,36,37,40,41,42] studies were included. They represented North America and Australia; while some studies on pediatric exposure to biomass smoke from South America and Asia were screened, they were excluded due to a focus on seasonal controlled agricultural biomass burns, not wildfires. Most of the included studies focused on a single city (Albuquerque, NM, USA [39], Sydney, AU [34], Darwin, AU [32], Valencia, SP [16]) or region (Northern California, USA [38], Hoopa Valley, California, USA [35], Southern California, USA [15,30,33,36], Colorado, USA [40], North Carolina, USA [42], Washington State, USA [31], Victoria, AU [41], British Columbia, Canada [23]); one evaluated 10 years of data across the United States, using US Centers for Disease Control data for medical visits [37]. Four were exclusively urban [16,32,34,39], one covered only an Indigenous reserve site [35] and the remainder included mixed urban and rural populations [15,23,30,31,33,36,37,38,40,41,42].

Data for 565,321 children under the age of 20 years was reported in 13 of the 16 included studies [15,16,23,30,31,33,35,36,38,39,40,42,43]; the remaining three did not report their results in terms of population numbers [32,37,41]. Only three studies evaluated preschool age categories separately [23,30,33]. Two studies surveyed existing pediatric cohorts (age 5 in Spain [16], ages 6–7 and 17–18 in Southern California [15]) regarding symptoms during wildfire smoke exposure; the others were population-level and relied on government and/or medical care provider system databases focusing on respiratory or cardiorespiratory causes for hospitalization or ED visit [23,30,31,32,33,35,36,37,38,39,40,41,42]. International Classification of Diseases and Related Health Problems, 9th Revision (ICD-9) codes were used to identify outcomes in all of the studies except one, which relied on self-report data [15]. Some were reported as “all respiratory visits”, while others also reported specific diagnostic codes including asthma, bronchiolitis, bronchitis, pneumonia and upper respiratory tract infection. Several studies included chronic obstructive pulmonary disease, with no pediatric cases recorded. The most reported outcomes were ED visits in eight studies [23,33,34,36,38,39,40,42], hospitalizations in four [30,31,32,33] and outpatient clinic visits in three [33,35]. One of two studies that captured individual symptoms also reported physician visits for smoke-related symptoms [15]. Three studies reported trends in healthcare presentations rather than OR or RR [32,37,41].

Different comparison groups, and different approaches to comparison between groups, were reported. Some before and after studies compared healthcare visits during the period immediately preceding and/or after a wildfire [15,16,30,32,33,38,39,41], others during previous months or years [23,35,36], in some cases matching the month and day of the week to days during the exposure period [23]. Fractures were assumed to be non-wildfire-associated injuries and used as a stable baseline for comparison to cardiorespiratory visits attributable to wildfire smoke exposure [31,42]. An alternative study design was to compare populations from a single medical data source by exposure status, typically designated by postal or zip code on the medical record between exposed and unexposed areas [37].

### 3.3. Exposure Characterization

Wildfire smoke exposure reporting differed between studies. Particulate matter was most common, with PM_2.5_ [30,31,33,34,36,37,38,39,40,41,42] or PM_10_ [15,23,32,35,41] measured mainly using locally deployed sampling devices, often through access to government- or agency-based air quality monitoring programs. Different types of system were used; studies that deployed their own air quality monitoring devices typically employed tapered element oscillating microbalance devices [32,35,41], although in the 1990s, gravimetric stacked filter units were also used [32]. Several studies added satellite data specifically focused on particulate matter analyses associated with wildfires [23,31,33,36,38,40]. Weather characteristics including temperature, barometric pressure and humidity were added to modeling methods employed in one study [30]. Other measures of exposure included ozone [37,38,41], visibility [32] and the Air Quality Index (AQI) [33], often as adjuncts within modeling algorithms that employed PM as a primary measure of exposure. Population exposure was determined by the postal or zip code on the medical record, although three studies used addresses [16,23,41], one county-level data [42] and one country-wide data [37]. Exposures were reported as a daily average in most studies [15,23,30,31,33,34,36,38,39,40,41,42]. Lag times between reported exposure and medical visit data varied; some studies evaluated same-day results [23,30,35,36] while others reported a range of lag times from 0 to 21 days after peak smoke events [15,31,32,33,38,39,41,42]. One reported only a lag time of three days [40] and two did not clarify the lag time between exposure and outcome [16,37]. In one of the studies, perceived symptoms were greater when the subjects reported being able to smell smoke, but no comparison of measured and perceived exposure level was reported [16].

### 3.4. Outcomes

Reported outcomes are summarized in Table 2. The most frequently reported outcome was ED visits for any respiratory cause (nine studies) [23,33,34,36,38,39,40,41,42] or for asthma (nine studies) [23,33,34,37,38,39,40,41,42]. Hospitalizations for asthma or any respiratory cause were reported in four studies [30,31,32,33], outpatient medical clinic visits in three [15,33,35] and symptoms in two [15,16].

### 3.5. Risk of Bias Assessment

**Figure 2:**
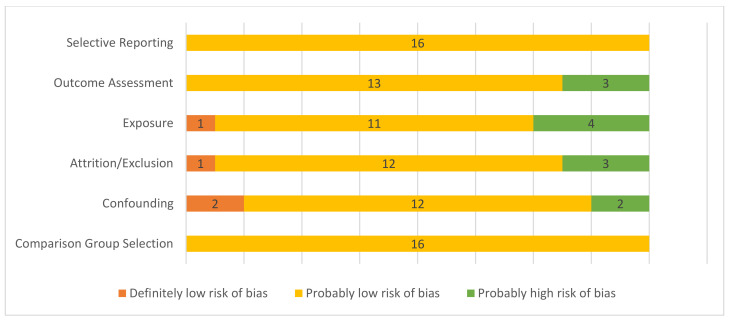
Risk of bias summary for included studies. Definitely low risk of bias = direct evidence of low risk-of-bias practices; probably low risk of bias = indirect evidence of low risk-of bias practices or deviations would not appreciably bias results; probably high risk of bias = indirect evidence of high risk-of-bias practices or insufficient information provided for evaluation [25,26].

A summary of the risk of bias assessment can be found in Figure 2. Of the 17 studies evaluated, one was determined to be at high risk of selection bias, adjusting for potential confounders, and exposure misclassification bias, so was excluded from the analysis. There was an increased risk of detection bias due to exposure characterization in three studies that used indirect means to estimate exposure: visibility index [32], although correlated with PM_10_ evaluation at a nearby site, and perceived exposure [15,16] and one estimated exposure based on modeling and did not adjust for seasonal trends [37]. There was a high risk of bias due to attrition in two population-level studies [32,35]: one because some residents, particularly those at high risk of respiratory disease, evacuated from the area [35], and in one cohort study [15] with limited response rates for the wildfire symptoms survey in a subset of the cohort. Two studies expressed data as trends [32,41]; however, the rest provided data with confidence intervals.

### 3.6. Association between Wildfire Smoke Exposure and Healthcare Visits

Health outcomes associated with wildfire visits are summarized in Table 3. Three studies encompassing 9977 participants noted a positive association between wildfire smoke exposure and outpatient clinic visits for any respiratory problem [15,33,35]; all were observational, and due to potential risk of bias the certainty of the evidence (GRADE) was low [28], whereas a larger number of participants (557,454 participants across 8 studies) demonstrated a positive association between respiratory visits to the ED and wildfire smoke exposure [23,33,36,38,39,40,42,44] with moderate certainty [28]. The four studies that demonstrated a positive association between hospitalization for any respiratory cause and wildfire smoke in the pediatric age group encompassed 13,258 participants [30,31,33,38], with moderate certainty and evidence of a dose effect [28]. The same studies showed a moderate certainty of evidence [28] in the positive association between ED visits and hospitalizations for asthma and exposure to wildfire smoke [23,30,31,33,34,36,38,39,40,42]. No significant association was noted for asthma-related clinic visits, with only one [33] of the three studies evaluating this outcome [15,16,33] showing a positive association and a low certainty of evidence [28].

### 3.7. Association between Wildfire Smoke Exposure and Symptoms

Only two studies assessed wildfire smoke-related symptoms [15,16]. There was a positive association between respiratory symptoms, with a very low certainty of evidence (GRADE) mainly due to risk of bias in exposure characterization and outcome assessment [28] in both studies and only one respiratory symptom (dry cough) being reported in one study [16]. Itchy or watery eyes, sneezing, sore throat and rhinitis had a positive association with smoke in both [15,16], with a dose effect and increased impact on participants with existing asthma or rhinitis reported in one study [16] and a low certainty of evidence [28].

### 3.8. Special Populations

Two studies addressed marginalized populations [32,35] and one specifically reported the difference between a marginalized Indigenous subpopulation and the total exposed group [32]. The impact on marginalized populations was not reported in one of the two studies, since the only non-Indigenous patients seen in the clinic during the wildfire period were firefighting personnel and only Indigenous patients typically attended the clinic in comparison periods [35]. One study did compare Indigenous to non-Indigenous participants, although this part of the study did not distinguish between children and adults. In this study, Indigenous patients were 15.02% (95% confidence interval 3.73%, 27.54%) more likely to be admitted to hospital for wildfire-attributable respiratory causes than non-Indigenous patients with the same level of PM_10_ exposure [32].

## 4. Discussion

### 4.1. Summary of Evidence

This review used a descriptive approach to summarize and evaluate the existing evidence on the impact wildfire smoke has on healthcare utilization in the pediatric population. All of the studies included in the review were observational, with either a pre–post or cross-sectional design. They encompassed urban or mixed urban and rural settings, other than one that focused on mainly rural exposures [42]. It was not possible to combine study data due to the significant heterogeneity in study design as well as differences between populations, comparison groups, exposures and outcomes [24]. Although outcome measures (healthcare visits, symptoms) were similar, there were differences in lag times between exposure and outcomes as well as reporting.

There is some evidence suggesting a positive association between wildfire smoke exposure and outpatient ED visits or hospitalizations for any respiratory diagnoses in the pediatric population, with no significant association specifically between asthma and ED visits. Eye itchiness, nasal congestion, rhinitis and sore throat were positively associated with wildfire smoke exposure with a low grade of certainty; respiratory symptoms such as wet or dry cough, asthma exacerbation, bronchitis or sneezing showed an increase that did not reach significance, with a very low grade of certainty (Table 3) [15,16]. Overall, there were no significant associations found between wildfire smoke exposure and pediatric outpatient visits or hospitalizations specifically for asthma (Table 2 and Table 3). It is possible that children with asthma spend more time indoors when air quality is poor and are more likely to increase their asthma medication proactively or in response to increased symptoms; in at least one of the included studies, higher-risk people were evacuated from the affected area [35].

Only two of the studies that recruited participants from an existing pediatric cohort focused solely on pediatric data [15,16]; the rest were population-level studies that included separately reported pediatric data but did not focus specifically on pediatric outcomes. In this context, four broke pediatric data into age-specific subgroups [23,30,33,36] while the rest reported results for participants < 15–20 years of age. Specifically in younger children, two studies found no significant association between wildfire smoke and respiratory ED visits [23,36]; however, the other two did note significant associations specifically in children less than 4 years of age, with no [30] or weaker [33] but significant associations between respiratory-related ED visits and wildfire smoke exposure in older children. While the two studies that specifically surveyed children from existing research cohorts did provide pediatric data, the age groups, total study numbers and outcomes were limited and using these pre-existing cohorts may have introduced selection bias. Given the significant differences in typical activities, airway size, respiratory reserve and developmental stage in young children [18], particularly in comparison with older children and adult-sized teens, this review highlights the paucity of existing data and the need for focused research on the response to wildfire smoke exposure in these very important age groups. As well, the importance of considering pediatric age groups as separate entities during population-level data analysis is evident from the studies that do show differences between infant, toddler, child and teen presentations [33].

Wildfire smoke composition is complex and dynamic [7]. The nature of exposure would be drastically variable within and across studies, as many factors including physical activity levels, length of time exposed, access to well-ventilated housing and weather trends all impact the true amount of exposure among participants. Few studies described the type of vegetation and other materials burned (e.g., houses, industrial materials and sites, type of trees and other plant matter), most only mentioning whether the smoke resulted from controlled, uncontrolled or mixed types of burning. The chemical composition of the smoke will vary depending on the material burned and the stage of burning, making it difficult to fully understand what chemical exposures were present across included studies.

More than half of the studies used PM_2.5_ as a primary measure of exposure; as a common component of air pollution whose role in pulmonary disease is well characterized, this is an option that should allow data from different studies to be compared and combined, provided other study characteristics are sufficiently similar. As well, PM_10_, another commonly measured component of air pollution that has already been associated with health outcomes, was employed by several more studies; while not directly comparable to PM_2.5_, reporting it in air pollution studies would likely be beneficial. Unfortunately, the means by which exposures were measured and reported, the length of time of exposure and lag time between exposure and outcome measurement varied considerably between studies. Other exposures that have been implicated in respiratory disease, including ozone, were also reported by some studies but not others. The heterogeneity between studies is likely at least in part due to differences in local measurement and reporting standards, since many relied on local, regional, or national air quality monitoring systems for information. Some studies added modeling to account for more detail in localizing which sites were impacted by wildfire smoke that included weather conditions and satellite imagery. While this may have improved accuracy in estimating exposed populations, it was not comparable between studies. Given the importance of air quality monitoring for studies evaluating any form of air pollution impacts on population health as well as increasing importance of and interest in monitoring exposures attributable to wildfire smoke, this review demonstrates that a simple universal system of exposure reporting, even if some studies also include more complex modeling, would promote data integration and collaborative efforts to understand the true impact of these exposures.

### 4.2. Challenges Associated with Synthesis

The study of exposure to wildfire smoke, like other exposure-related studies, tends to rely on observational data. Although some areas frequently impacted by wildfires can potentially be prepared for a more rigorous study design, for the most part it would not be feasible to entertain alternate research options. Study design is often influenced by the availability of data, including population and exposure characterization. Pooling data and meta-analysis were not possible in this review due to heterogeneity in study design, exposure characterization, population and comparison group definition, outcome assessment and reporting. Additionally, the lack of information available regarding pre-existing conditions among participants that may have affected the outcomes of the included studies limits the interpretation of these results.

### 4.3. Strengths and Limitations

We followed the Cochrane guidelines for conducting a systematic review and PRISMA reporting guidelines [20,24,45]. A detailed protocol outlining methodology, data extraction and data synthesis was published in advance of the review on PROSPERO. Comprehensive searches conducted by a health sciences librarian identified literature discussing pediatric healthcare utilization associated with wildfires. Two independent reviewers conducted the screening and risk of bias assessments as per the Cochrane guidelines [45]. These are all strengths of this study that added thoroughness to the approach used to detect eligible research studies and rigor in selecting them. However, there were also limitations. This review is limited by an inability to complete a quantitative analysis or accurate calculation of risk of bias due to the large heterogeneity in study design across included studies [24]. The review focused specifically on wildfires, excluding prescribed vegetation burns, to decrease heterogeneity between types of exposure; however, this excluded some studies with mixed contributors to smoke, as well as particularly excluding several studies from Asia and Brazil, where yearly prescribed burning was identified as a significant contributor to overall smoke days and smoke-related PM_2.5_ impacting air quality [43]. Few studies reported pediatric outcomes, and the small number of studies that broke down data by age did not allow for adequate comparison between infants, preschool children and teenagers, although physiologically and developmentally they are distinct populations. In one study, original data were not available from the publication or from the author, so numbers were estimated based on measuring points and confidence intervals on a graph in the original publication [23], which may have impacted the results.

## 5. Conclusions

A limited number of observational studies available in the literature suggest that children have an increased risk of respiratory-related healthcare visits associated with wildfire smoke exposure. With the increasing quantity and severity of wildfires in some regions, it is imperative to investigate the respiratory health implications of wildfire smoke at both an individual and population level. Future research should include longitudinal observational studies investigating the long-term impact of wildfire smoke exposure on children, as well as break down the impact of exposure by age. Additionally, promoting a standard means for reporting wildfire smoke exposures and outcomes will promote data integration in the future.

## Figures and Tables

**Table 1:** Characteristics of included studies. Values that reach significance are in bold.

Study	Trial Characteristics	Population Characteristics	“Smoke Event” Days	Comparison Group	Measurement Characteristics	Outcomes ED Visits	Outcomes: Hospitalizations	Outcomes: Symptoms	Outcomes: Outpatient Visits
Delfino et al., 2009 [30] Site: Southern California, USA Funding: South Coast Air Quality Management District and NIH research grant	Design: Pre/post Enrollment: October 1–20 (pre-fire), 21–30 October (during the fire), 31 October–15 November (post-fire) 2003 Setting:	N: 3374 Age: <19 years Data source: Office of Statewide Health Planning and Development (OSHPD) Other: None	Definition: All cardiorespiratory hospital admissions Number of days: 10 Exposed population: Zip code during the exposure period	Population: All patients admitted to hospital in California state Comparator: Periods directly before and after wildfire smoke peak in 2003	PM_2.5_: per 10 mg/m^3^ increase in PM_2.5_ PM_10_: Not reported Other measure: Temperature, humidity, surface pressure gradient Monitoring: governmental network site data Time between exposure and outcome: no lag time	Not reported	Age 0–4 wildfire period All resp **RR 1.05 (1.01–1.08)** Asthma **RR 1.08 (1.02–1.15)** Acute bronchitis/bronchiolitis RR 1.09 (1.00–1.20) Acute Pneumonia RR 1.02 (0.95–1.09) post-wildfire period All resp RR 0.89 (0.81–0.99) Asthma RR 0.92 (0.77–1.11) bronchitis/bronchiolitis RR 1.03 (0.87–1.22) Pneumonia RR 0.82 (0.65–1.04) Age 5–19 wildfire period All resp RR 1.03 (0.98–1.08) Asthma RR 1.00 (0.94–1.07) Pneumonia RR 1.06 (0.99–1.14) post-wildfire period All resp RR 0.96 (0.85–1.08) Asthma RR 0.92 (0.79–1.07) Pneumonia RR 1.02 (0.77–1.35)	Not reported	Not reported
Gan et al., 2017 [31] Site: Washington State, USA Funding: Research grant	Design: Cross-sectional Enrollment: 1 July–31 October 2012 Setting: Urban and rural	N: 1304 Age: <15 years Data source: Washington State department of Health Comprehensive Hospital Abstract Reporting System (CHARS) Other: limited to emergency or urgent care	Definition: respiratory ED and urgent care visits Number of days: 123 Exposed population: wildfire smoke exposure by zip code	Population: respiratory ED and urgent care visits associated with air pollution attributed to wildfire smoke Comparator: Fracture of radius and ulna in the same population during the same time period	PM_2.5_: >10 mg/m^3^ = smoke day PM_10_: not reported Other measure: not reported Monitoring: Weather Research and Forecasting with Chemistry (WRF-Chem) and Geographically Weighted Regression (GRF) (aerosol optical depth modelling) Time between exposure and outcome: 0 to 5 days prior to admission	Not reported	GWR Smoke All resp OR 1.07 (1.00–1.14) WRF-Chem Smoke All resp **OR 1.06 (1.01–1.11)** Asthma **OR 1.11 (1.05–1.18)**	Not reported	Not reported
Hanigan et al., 2008 [32] Site: Darwin, Australia Funding: Research grant and Norther Territory Government and Bureau of Meteorology	Design: Cross-sectional Enrollment: 1 April to 30 November 1996–2005 Setting: Urban	N: not reported Age: <15 years Data source: Northern Territory Department of Health and Community Services Other: Indigenous status for subgroup analysis	Definition: respiratory ED visits and hospital admissions for children on wildfire smoke impacted days Number of days: 2410 dry season days over 10 years; not all smoke days Exposed population: children in Darwin on smoke days	Population: children living in Darwin on smoke days Comparator: the same population on non-smoke days	PM_2.5_: not reported PM_10_: estimates based on visibility measures Other measure: none reported Monitoring: in 2000, PM_10_ was measured using a Tapered Element Oscillating Microbalance; in 1995 a gravimetric stacked filter unit was used Time between exposure and outcome: No lag and 1–3 day lags	Data not expressed as OR, RR, or excess visit “% < 15 years for each category (proportional)” Positive trend in respiratory admissions with same-day increased PM_10_ for respiratory infections and asthma with no breakdown for <15 years			
Henderson et al., 2011 [23] Site: British Columbia, Canada Funding: Research grants	Design: Cross-sectional Enrollment: 1 July to 30 September 2003 Setting: Mixed rural and urban	N: 60,848 Age: <20 years Data source: British Columbia Medical Services Plan Other: none	Definition: All respiratory claims by date Number of days: 92 Exposed population: By physical address, corroborated by postal code in medical file	Population: all British Columbia Medical Services Plan users in the study period, based on the postal code data being up to date within a year before or after the study period Comparator: year before and year after; three different categories of smoke exposure	PM_2.5_: not measured PM_10_: mean exposure 29 Other measure: satellite estimates of exposure and PM_10_ Monitoring: Tapered element oscillating microbalance and satellite images Time between exposure and outcome: no lag	All outcomes day of peak exposure: Age 0–4 All respiratory outcomes OR 1.02 (0.97–1.07) Age 5–9 All respiratory outcomes OR 0.95 (0.89–1.01) Age 10–19 All respiratory outcomes OR 1.03 (0.99–1.07) * OR estimated by manually measuring graphically reported results in the manuscript; the authors no longer retained original data ** All health care visit types combined			
Hutchinson et al., 2018 [33] Site: California, USA Funding: Research grants	Design: Cross-sectional Enrollment: 16 August to 15 December 2007; three fire periods P1: 22–26 October, P2: 27–31 October and P3: 1–3 November 2007 Setting: Mixed rural and urban	N: 7932 Age: 0–17 Data source: Medi-Cal administrative claims data form the California Department of Health Care Services (DHCS) Management Information System/Decision Support System (MIS/DSS), San Diego County Other:	Definition: All cardiorespiratory Medi-Cal claims for outpatient and ED visits and hospitalizations (first day within 14 days of fire period) Number of days: 15 Exposed population: zip code during the exposure period	Population: all patients with Medi-Cal claims during the enrollment period Comparator: Periods starting 3, 4, 5, 6, 8 and 9 weeks before each exposure period and all-cause (total) visits for each healthcare setting to provide context for outcomes of interest	PM_2.5_: 24 h mean P1 89.1 mg/m^3^, P2 9.33 mg/m^3^, P3 0.26 mg/m^3^ PM_10_: not reported Other measure: Air Quality Index (AQI) Monitoring: Geospatial tool Wildland Fire Emissions Information System (WFEIS) Time between exposure and outcome: no lag time (P1 no lag, P2 low exposure and lag, P3 lag and cumulative effects)	All outcomes 5 days peak exposure Age 0–1 All resp index **RR 1.77 (1.15–1.66)** Asthma **RR 3.43 (1.49–7.38)** Acute Bronchitis **RR 2.95 (1.15–6.85)** Bronchitis RR 0.00 (0.00–6.57) Pneumonia RR 0.84 (0.13–3.12) URI **RR 1.82 (1.25–2.66)** Respiratory Symptoms **RR 2.06 (1.33–3.22)** Age 2–4 All resp index RR 1.50 (0.91–2.48) Asthma RR 1.39 (0.41–3.76) Acute Bronchitis RR 1.33 (0.06–9.03) Bronchitis RR 2.00 (0.08–15.92) Pneumonia RR 1.14 (0.05–7.39) URI RR 1.63 (0.85–3.12) Respiratory Symptoms RR 1.45 (0.55–3.31) Age 0–4 All resp index **RR 1.70 (1.32–2.19)** Asthma **RR 2.36 (1.27–4.39)** Acute Bronchitis **RR 2.56 (1.09–5.54)** Bronchitis RR 0.89 (0.04–5.41) Pneumonia RR 0.92 (0.22–2.76) URI **RR 1.77 (1.28–2.45)** Respiratory Symptoms **RR 1.91 (1.29–2.82)** Age 5–17 All resp index RR 1.19 (0.80–1.79) Asthma **RR 2.00 (1.09–3.67)** Acute Bronchitis RR 1.78 (0.26–7.46) Bronchitis RR 0.00 (0.00–8.92) Pneumonia RR 1.45 (0.22–5.85) URI RR 1.03 (0.46–2.07) Respiratory Symptoms RR 1.14 (0.62–2.09)	All outcomes 5 days peak exposure Age 0–1 All resp index RR 0.99 (0.46–1.90) Asthma RR 2.18 (0.49–7.27) Acute Bronchitis RR 1.78 (0.26–7.46) Pneumonia RR 1.26 (0.30–3.90) URI RR 1.50 (0.35–4.74) Respiratory Symptoms RR 0.86 (0.26–2.24) Age 2–4 All resp index RR 2.13 (0.61–6.11) Asthma RR 3.43 (0.72–13.05) Acute Bronchitis RR 0.00 (0.00–13.72) Pneumonia RR 2.67 (0.10–25.02) URI RR 0.00 (0.00–6.57) Respiratory Symptoms RR 1.33 (0.06–9.03) Age 0–4 All resp index RR 1.18 (0.66–2.12) Asthma RR 2.67 (0.97–6.53) Acute Bronchitis RR 1.33 (0.20–5.28) Pneumonia RR 1.45 (0.43–3.95) URI RR 1.14 (0.27–3.49) Respiratory Symptoms RR 0.93 (0.33–2.20) Age 5–17 All resp index RR 1.13 (0.53–2.18) Asthma RR 0.76 (0.12–1.18) Acute Bronchitis RR 0.00 (0.00–13.72) Pneumonia RR 0.64 (0.10–2.31) URI RR 0.00 (0.00–5.18) Respiratory Symptoms RR 1.74 (0.59–4.35)	Not reported	All outcomes 5 days peak exposure Age 0–1 All resp index RR 1.09 (0.99–1.2) Asthma **RR 1.54 (1.11–2.12)** Acute Bronchitis **RR 1.63 (1.21–2.19)** Bronchitis RR 1.17 (0.45–2.62) Pneumonia RR 1.55 (0.95–2.54) URI RR 1.00 (0.89–1.12) Respiratory Symptoms **RR 1.34 (1.02–1.76)** Age 2–4 All resp index RR 1.13 (1.00–1.28) Asthma RR 1.18 (0.91–1.55) Acute Bronchitis RR 0.94 (0.37–2.08) Bronchitis RR 0.57 (0.09–2.04) Pneumonia RR 1.55 (0.93–2.56) URI RR 1.04 (0.88–1.21) Respiratory Symptoms **RR 1.75 (1.28–2.39)** Age 0–4 All resp index **RR 1.11 (1.03–1.19)** Asthma **RR 1.31 (1.07–1.61)** Acute Bronchitis **RR 1.52 (1.15–2.00)** Bronchitis RR 0.93 (0.42–1.85) Pneumonia **RR 1.55 (1.09–2.20)** URI RR 1.01 (0.92–1.11) Respiratory Symptoms **RR 1.49 (1.22–1.84)** Age 5–17 All resp index RR 0.93 (0.83–1.05) Asthma RR 1.25 (1.05–1.48) Acute Bronchitis **RR 1.25 (0.64–2.43)** Bronchitis RR 1.64 (0.72–3.39) Pneumonia RR 0.75 (0.32–1.54) URI RR 0.70 (0.59–0.84) Respiratory Symptoms RR 0.93 (0.64–1.36)
Johnston et al., 2014 [34] Site: Sydney, Australia Funding: Research grants, government	Design: Cross-sectional (case-crossover) Enrollment: 1 July 1996–30 June 2007 Setting: Metropolitan area	N: 344,933 (52% of 663,333 were pediatric) Age: <15 years Data source: NSW Emergency Department Data Collection Other: ICD codes for respiratory, cardiac and cerebrovascular ED visits	Definition: Citywide PM > 99th percentile (44/46 days wildfire, 2/46 days prescribed burn) Number of days: 46 Exposed population: by postal code	Population: same individuals Comparator: Matched non-smoke days in the same year, month and day of the week	PM_2.5_: mean 39.1 (non-smoke 9.9) mg/m^3^ PM_10_: mean 60.5 (non-smoke 17.8) mg/m^3^ Other measure: not reported Monitoring: Government air quality stations Time between exposure and outcome: Lag 0, 1, 2, 3 days	Lag 0 All Resp OR: 1.01 (0.97–1.06) Asthma OR 1.06 (0.97–1.17) Pneumonia/Bronchitis OR 0.96 (0.85–1.07) Lag 1 All Resp OR 1.00 (0.96–1.05) Asthma OR 1.05 (0.96–1.15) Pneumonia/Bronchitis OR 0.97 (0.87–1.09) Lag 2 All Resp OR 0.94 (0.90–0.98) Asthma OR 0.97 (0.89–1.07) Pneumonia/Bronchitis OR 1.05 (0.94–1.18) Lag 3 All Resp OR 0.97 (0.93–1.01) Asthma OR 1.00 (0.91–1.09) Pneumonia/Bronchitis OR 1.01 (0.90–1.13)	Not reported	Not reported	Not reported
Kunzli et al., 2006 [15] Site: Southern California Funding: Data provision by South Coast Air Quality Management District 041829	Design: Cross-sectional (case-crossover) survey of an existing cohort Enrollment: 20 October to 3 November 2003 Setting: Mixed rural and urban	N: 834 age 17–18; 3775 age 6–7; 4609 total Age: 6–7 and 17–18 years Data source: Questionnaires Other: Participants were recruited from the existing University of California Children’s Health Study (CHS) (Kunzli)	Definition: Population exposed to the fire or smoke from the fire Number of days: not described Exposed population: by address (survey of existing cohort); 35 participants lost their home in the fire	Population: children enrolled in the University of California Children’s Health Study Comparator: Same population before the fire	PM_2.5_: not reported PM_10_: 2, 3, 4 or 5-day mean PM_10_ level depending on length of fire smoke exposure of a community Other measure: “smell of fire smoke” indoors Monitoring: local air quality monitors, Time between exposure and outcome: 1–2 days, 3–5 days, ≥6 days	Not reported	Not reported	1–5 days smoke smell Itchy/Watery eyes **OR 2.26 (1.90–2.68)** Irritated eyes **OR 2.38 (2.01–2.82)** sneezing/nasal symptoms **OR 1.98 (1.68–2.33)** cold **OR 1.50 (1.25–1.81)** sore throat **OR 1.81 (1.53–2.14)** dry cough at night **OR 2.25 (1.87–2.71)** dry cough in morning **2.24 (1.85–2.72)** dry cough other times **OR 2.67 (2.20–3.24)** wet cough **OR 1.42 (1.13–1.79)** wheezing or whistling **OR 2.15 (1.63–2.83)** wheeze/disturb sleep **OR 2.29 (1.56–3.37)** wheeze/limit speech **OR 2.23 (1.03–4.83)** asthma attack OR 1.32 (0.84–2.07) bronchitis OR 1.33 (0.87–2.02) medication for above **OR 1.82 (1.51–2.19)** miss school for above **OR 1.59 (1.25–2.02)** >6 days smoke smell Itchy/Watery eyes **OR 4.11 (3.36–5.02)** Irritated eyes **OR 4.42 (3.61–5.41)** sneezing/nasal symptoms **OR 2.79 (2.30–3.39)** cold **OR 2.13 (1.73–2.63)** sore throat **OR 2.50 (2.05–3.05)** dry cough at night **OR 3.35 (2.71–4.15)** dry cough in morning **OR 2.91 (2.33–3.63)** dry cough other times **OR 3.27 (2.61–4.09)** wet cough **OR 2.15 (1.67–2.77)** wheezing or whistling **OR 3.53 (2.62–4.75)** wheeze/disturb sleep **OR 4.94 (3.33–7.33)** wheeze/limit speech **OR 5.49 (2.63–11.48)** asthma attack OR 1.63 (1.00–2.67) bronchitis **OR 2.23 (1.45–3.43)** medication for above **OR 2.33 (1.89–2.88)** miss school for above **OR 2.24 (1.72–2.91)**	1–5 days smoke smell visit doctor for symptoms **OR 1.33 (1.02–1.74)** >6 days smoke smell visit doctor for symptoms **OR 2.03 (1.53–2.71)**
Lee et al., 2009 [35] Site: Hoopa Valley, California, USA Funding: None reported	Design: Pre/post Enrollment: 17 August to 4 November 1999 Setting: Indigenous Reserve/Rural	N: 1211 Age: <19 years Data source: Hoopa Reservation Medical Clinic electronic medical record Other: Patients with unknown residence were excluded	Definition: Population exposed to wildfire smoke was presumed by presenting to the Hoopa Valley Medical Centre Number of days: 84 Exposed population: Residents with addresses from Hoopa Valley, Burnt Ranch, Salyer Area, Weitchpec and Willow Creek Area and non-residents (presumed to be firefighters deployed to the area)	Population: All individuals who visited the Hoopa Valley Medical Centre Clinic for cardiorespiratory outcomes Comparator: Same calendar days in the previous year (1998)	PM_2.5_: Not reported PM_10_: Maximum daily PM_10_ in 1999 were 619.8 mg/m^3^ and in 1998 were 175 mg/m^3^ Other measure: not reported Monitoring: Hoopa’s Tribal Environmental Protection Agency used a tapered element oscillating microbalance ambient particulate monitor for hourly measurements Time between exposure and outcome: no lag time	Not reported	Not reported	Not reported	All resp clinic visit Resident in fire zone **OR 1.74 (1.24–2.43)** Resident nearby fire zone OR 0.86 (0.26–2.81) Non- resident OR 2.99 (0.33–26.90) Asthma clinic visits OR 1.39 (0.77–2.51)
Leibel et al., 2020 [36] Site: San Diego County, California, USA Funding:	Design: Cross-sectional Enrollment: 6–17 December 2017 Setting: Mixed rural and urban	N: 30,087 Age: <19 years; subdivided into 0–5, 6–12, 13+ years Data source: Rady Children’s Hospital and University of California Clinics Electronic Medical Record Other: None	Definition: Exposed population residing in San Diego County based on zip code Number of days: 12 Exposed population: Zip code during the exposure period	Population: Patients visiting the Rady Children’s Hospital and University of California pediatric clinics Comparator: Same weeks (during same calendar month) in 2011 to 2016	PM_2.5_: average daily increase of 5.6 mg/m^3^ PM_10_: Not reported Other measure: Correlated with satellite imaging from the Moderate Resolution Imaging Spectroradiometer (MODIS) Rapid Response System Monitoring: US EPA Air Quality System from San Diego County Time between exposure and outcome: No lag time	Age 0–5 All resp ED excess visit 7.30 (3.00–11.70) All resp Urgent Care excess visit 7.7 (4.1–11.3) Age 6–12 All resp ED excess visit 3.40 (2.30–4.60) All resp Urgent Care excess visit 3.60 (2.30–4.90) Age 13–19 All resp ED excess visit 2.00 (1.00–3.00) All resp Urgent Care excess visit 3.30 (2.30–4.20) All Ages (0–19) All resp ED excess visit 16.00 (11.60–21.60) All resp Urgent Care excess visit 16.60 (11.60–21.60)	Not reported	Not reported	Not reported
Pratt et al., 2019 Site: USA [37] Funding: None reported	Design: Cross-sectional Enrollment: May to September 2005–2014 Setting: Mixed rural and urban (country-level data)	N: not reported Age: 0–18 years Data source: No primary data; secondary estimates based on US Centers for Disease Control reported values for children with asthma Other: Behavioral Risk Factor Surveillance System (BRFSS; US Centers for Disease Control), National Health Interview Survey (US Centers for Disease Control) to estimate the number of children with asthma	Definition: ED visits for asthma in children attributable to wildfire smoke exposure Number of days: not reported Exposed population: Children living in areas where ozone and PM_2.5_ were elevated by ≥1 standard deviation above the local mean based on nearest US Environmental Protection Agency measuring device	Population: Median ED visits per 100,000 children in the presence of wildfire smoke Comparator: Median ED visits per 100,000 children in the absence of wildfire smoke exposure	PM_2.5_: ≥1 standard deviation > the station mean PM_10_: not reported Other measure: Elevated ozone attributable to wildfire smoke (EOAS); smoke present in the atmospheric column Monitoring: Time between exposure and outcome: not reported	Data not expressed as OR, RR, or excess visit “overall median estimated ED visit in children with asthma that may be attributed to EOAS for the study period” The number of visits attributed to EOAS was 2403 (95% CB 235–5383) ED visits			
Reid et al., 2016 [38] Site: Northern California: Sacramento valley, san Francisco Bay Area, mountain Counties, Lake County, North Central Coast, northern San Joaquin Valley Funding: Research grants, US EPA	Design: Pre/post Enrollment: Pre-fire 6 May–19 June 2008 (43 days); Fire period 20 June to 31 July 2008 (42 days), Post-fire period 1 August to 15 September 2008 (46 days) Setting: Mixed rural and urban	N: 10,363 (ED visits); 648 (hospitalizations) Age: <20 years Data source: Office of Statewide Health, Planning and Development (California, USA) Other: Hospital admission and ED visits (OSHPD)	Definition: smoke attributable to wildfire Number of days: 42 Exposed population: by zip code during fire period	Population: Cross-sectional review of ED visits and hospitalizations by age on each day of exposed and comparison periods Comparator: Pre-fire period	PM_2.5_: Before 6.3, during 19.1, after 8.5 mg/m^3^ PM_10_: Not reported Other measure: Ozone before 54.4, during 47.6, after 60.0 ppb Monitoring: Modelling including 112 monitoring stations and aerosol optic depth from Geostationary Operational Environmental Satellite Time between exposure and outcome: same-day, 1 and 2 days after exposure	During fire All resp RR 0.99 (0.98–1.00) Asthma RR 1.03 (1.00–1.05) Pneumonia RR 0.98 (0.94–1.01)	During fire All resp RR 0.99 (0.96–1.03) Asthma RR 1.01 (0.94–1.09) Pneumonia RR 1.01 (0.96–1.07)	Not reported	Not reported
Resnick et al., 2015 [39] Site: Albuquerque, New Mexico, USA Funding: US Centers for Disease Control	Design: Pre/post Enrollment: 1 May to 8 July 2011 Setting: Urban	N: 1369 Age: <19 years Data source: New Mexico Department of Health Other: None	Definition: Population exposed to wildfire smoke Number of days: 13 Exposed population: Individuals residing in the Albuquerque area who reported to the ED for cardiorespiratory visits during the exposure period	Population: Patients visiting an Albuquerque-area ED for cardiorespiratory visits Comparator: Daily average ED visits during periods with no acute exposure	PM_2.5_: 24-h averages: pre-fire mean 6.8 mg/m^3^, acute mean 31.3 mg/m^3^, post-acute mean 14.5 mg/m^3^ PM_10_: not reported Other measure: AQI (comparator) Monitoring: City of Albuquerque air quality monitors (2), hourly recordings Time between exposure and outcome: No lag time reported	12 days during fire All resp RR 0.70 (0.61–0.82) Asthma RR 1.02 (0.74–1.30) Other resp RR 1.24 (0.62–2.50) 3 weeks post peak smoke All resp RR 0.54 (0.48–0.62) Asthma RR 0.79 (0.59–1.04) Other resp RR 0.75 (0.39–1.47)	Not reported	Not reported	Not reported
Stowell et al., 2019 [40] Site: Colorado, USA Funding: Research grants, US EPA	Design: Cross-sectional (case-crossover) Enrollment: May-August 2011 to May-August 2014 Setting: Statewide (urban and rural)	N: 94,022 Age: 0–18 Data source: Colorado Department of Public Health and Environment Other: ED visits and hospitalizations by ICD-9 code	Definition: smoke attributable to wildfire Number of days: not reported Exposed population: by 1 km^2^ spatial grid (exposure) and zip code	Population: same individuals Comparator: Four non-smoke days per smoke day per individual on the same day of the week and calendar month	PM_2.5_: Wildfire PM_2.5_ minus daily PM_2.5_ means; 0–37 mg/m^3^ PM_10_: Not reported Other measure: not reported Monitoring: Combined satellite and US EPA ground monitors Time between exposure and outcome: Lag 2 days cardiac, 3 days respiratory presentations	3 day average All Resp OR 1.02 (1.00–1.03) Asthma **OR 1.08 (1.04–1.12)** Bronchitis OR 0.97 (0.89–1.06) URI OR 1.01 (0.99–1.03)	Not reported	Not reported	Not reported
Tham et al., 2009 [41] Site: Victoria, Australia Funding: Post-doctoral fellowship	Design: Cross-sectional Enrollment: 1 October 2002–1 April 2003 Setting: Mixed rural and urban	N: not reported Age: <15 years Data source: Victorian Department of Human Services Other:	Definition: respiratory ED visits and hospital admissions for children on wildfire smoke impacted days Number of days: Not reported Exposed population: Children living in the area on smoke-exposed days based on elevated PM_10_, PM_0.1–1_ and ozone	Population: respiratory ED visits and hospital admissions on smoke-exposed days Comparator: respiratory ED visits and hospital admissions for the same location on non-smoke days	PM_2.5_: not reported PM_10_: 24-h averages Other measure: API (PM_0.1–1_) and ozone Monitoring: Tapered element oscillating microbalance, Airborne Particle Index and chemiluminescence for ozone Time between exposure and outcome: No lag and 1-day lag	Data not expressed as OR, RR, or excess visit “daily ED and hospital admissions”. A trend toward increased hospital admissions and ED visits on days with elevated wildfire smoke was not broken into individual age groups and did not reach significance.			
Tinling et al., 2016 [42] Site: North Carolina, USA Funding: No identified funding	Design: Cross-sectional Enrollment: May 5 to June 18 2011 Setting: Mixed rural and urban	N: 7900 Age: <18 years Data source: North Carolina Disease Event tracking and Epidemiologic Collection Tool (NCDETECT) Other: None	Definition: Exposed population residing in North Carolina by county Number of days: 45 Exposed population: County-level daily exposures to wildfire PM_2.5_	Population: All ED visits for cardiorespiratory outcomes Comparator: ED visits for bone fractures (not anticipated to have any wildfire-related changes)	PM_2.5_: Peak exposure days > 100 mg/m^3^ PM_10_: not reported Other measure: not reported Monitoring: Smoke Forecasting System (National Air Resources Laboratory of the National Oceanic and Atmospheric Administration) Time between exposure and outcome: 0- and 2- day lag time from exposure	Lag day 0–2 All resp ED visit **RR 1.09 (1.01–1.17)** Asthma RR 0.97 (0.86–1.09) URI **RR 1.14 (1.04–1.24)** Respiratory/other chest symptoms visit **RR 1.18 (1.06–1.33)**	Not reported	Not reported	Not reported
Vicedo-Cabrera et al., 2016 [16] Site: Valencia, Spain Funding: Research Grant	Design: Cross-sectional survey of an existing cohort Enrollment: 16–27 June; 28 June–8 July 2012 Setting: Mixed rural and urban including cities and villages	N: 496 Age: 5 Data source: The Infancia y Medio Ambiente (INMA) Project, Spain (Valencia Cohort) https://www.proyectoinma.org/ (accessesd on 19 August 2021) Other: Participants were recruited from an existing cohort to report on wildfire exposure symptoms	Definition: Population exposed to wildfire smoke Number of days: 11 Exposed population: Based on postal code and individual report of being present at that address during the smoke period	Population: 5 year old children enrolled in the INMA Project Comparator: Same population in the 11 day period immediately before the fire	PM_2.5_: not reported PM_10_: not reported Other measure: self-reported “perception of exposure” Monitoring: not reported Time between exposure and outcome: not reported	Not reported	Not reported	Overall Itchy/Water eyes **OR 3.11 (1.62–5.97)** Sneezing OR 1.39 (0.76–2.54) Sore throat **OR 3.02 (1.41–6.44)** Dry cough OR 1.29 (0.64–2.59) Smoke smell outdoors at least 1 day Itchy/Watery eyes **OR 3.53 (1.79–6.98)** Sneezing OR 1.38 (0.73–2.61) Sore throat **OR 3.20 (1.47–6.98)** Dry cough OR 1.41 (0.68–2.94) Smoke smell indoors at least 1 day Itchy/Watery eyes **OR 3.45 (1.60–7.44)** Sneezing OR 1.51 (0.67–3.38) Sore throat **OR 4.21 (1.76–10.05)** Dry cough OR 1.68 (0.69–4.11) Dense air outdoors at least 1 day Itchy/Watery eyes **OR 3.84 (1.76–8.35)** Sneezing OR 1.91 (0.87–4.22) Sore throat **OR 4.23 (1.78–10.04)** Dry cough OR 2.03 (0.85–4.85) Distance to fires >30 km Itchy/Watery eyes **OR 2.85 (1.47–5.51)** Sneezing OR 1.47 (0.79–2.72) Sore throat **OR 3.28 (1.48–7.28)** Dry cough OR 1.39 (0.69–2.83) Distance to fires ≤30 km Itchy/Watery eyes OR 2.06 (0.77–5.53) Sneezing OR 2.06 (0.66–6.43) Sore throat **OR 4.61 (1.43–4.88)** Dry cough OR 1.57 (0.46–5.36) Children without Rhinitis Itchy/watery eyes **OR 3.23 (1.58–6.59)** Sneezing OR 1.22 (0.66–2.27) Sore throat **OR 2.56 (1.18–5.55)** Dry cough OR 1.15 (0.55–2.42) Children with Rhinitis Itchy/watery eyes **OR 8.06 (1.98–32.88)** Sneezing **OR 7.19 (1.34–38.58)** Sore throat OR 2.48 (0.39–5.91) Dry cough OR 3.08 (0.49–19.33) Children without Asthma Itchy/watery eyes in **OR 3.23 (1.63–6.40)** Sneezing OR 1.33 (0.72–2.46) Sore throat **OR 2.81 (1.30–6.05)** Dry cough OR 1.29 (0.64–2.60) Children with Asthma Itchy/watery eyes **OR 9.26 (2.14–40.12)** Sneezing **OR 11.40 (2.01–4.52)** Sore throat **OR 6.25 (1.14–34.30)** Dry cough OR 3.93 (0.63–24.62)	Not reported

NA = not applicable, PM = particulate matter, OR = Odds Ratio, RR = Risk Ratio, AQI = Air Quality Index.

**Table 2:** Effect direction plot (sorted by alphabetical order).

Study	Study Design	Risk of Bias Issues	Wildfire Measure	Age (Years)	Respiratory Outcomes: Effect Direction					
					ED or Clinic Visits		Hospitalizations		Symptoms	
					RV	AV	RV	AV	Respiratory	Other
Delfino et al., 2009 [30]	Pre-post	NA	PM_2.5_, humidity, temperature	0–4 5–19	NR	NR	⇑ ◊	◊ ◊	NR	NR
Gan et al., 2017 [31]	Cross-sectional	NA	PM_2.5_	<15	NR	NR	⇑	⇑	NR	NR
Hanigan et al., 2008 [32]	Cross-sectional	Confounding, attrition, exposure characterization	Visibility index	<15	NR	NR	⇑	⇑	NR	NR
Henderson et al., 2011 [23]	Cross-sectional	NA	PM_10_, satellite	0–4 5–9 10–19	◊ ◊ ◊	NR	NR	NR	NR	NR
Hutchinson et al., 2018 [33]	Cross-sectional	NA	PM_2.5_, AQI	0–4 5–17	⇑ ◊	⇑ ⇑	◊ ◊	◊ ◊	NR	NR
Johnston et al., 2014 [34]	Cross-sectional	NA	PM_2.5_, PM_10_	<15	◊	◊	NR	NR	NR	NR
Kunzli et al., 2006 [15]	Cross-sectional	Attrition, outcome assessment	PM_10_, smell of fire smoke		⇑	◊	NR	NR	⇑	◊
Lee et al., 2009 [35]	Pre-post	Exposure characterization, attrition	PM_10_	<19	⇑	◊	NR	NR	NR	NR
Leibel et al., 2020 [36]	Cross-sectional	Outcome assessment	PM_2.5_, satellite	<19	⇑	NR	NR	NR	NR	NR
Pratt et al., 2019 [37]	Cross-sectional	Exposure characterization	PM_2.5_, ozone	0–18	NR	⇑	NR	NR	NR	NR
Reid et al., 2016 [38]	Pre-post	NA	PM_2.5_, ozone	<20	◊	◊	NR	NR	NR	NR
Resnick et al., 2015 [39]	Pre-post	Confounding variables	PM_2.5_, AQI	<19	⇓	◊	NR	NR	NR	NR
Stowell et al., 2019 [40]	Cross-sectional	NA	PM_2.5_	0–18	◊	⇑	NR	NR	NR	NR
Tham et al., 2009 [41]	Cross-sectional	NA	PM_10_, PM_2.5_, ozone	<15	◊	NR	NR	NR	NR	NR
Tinling et al., 2016 [42]	Cross-sectional	NA	PM_2.5_	<18	⇑	◊	NR	NR	NR	NR
Vicedo-Cabrera et al., 2016 [16]	Cross-sectional	Exposure characterization, outcome assessment	Self-reported exposure	5	NR	NR	NR	NR	◊	⇑

NA = not applicable, PM = particulate matter, RV = respiratory visits, AV = asthma visits, ED = emergency department, AQI = Air Quality Index. Effect direction: ◊ = no significant association, ⇑ = positive association, ⇓ = negative association, NR not reported All health care outcomes combined, including hospitalizations, ED and clinic visits.

**Table 3:** Summary of findings with certainty of evidence (GRADE [28]).

Outcomes	Effect ^a^	Number of Participants (Number of Studies)	Certainty of Evidence (GRADE) [28]
Outpatient clinic visits for any respiratory cause	All three studies observed a positive association between wildfire smoke and clinic visits for respiratory problems	9977 (3) [15,33,35]	Low All observational studies, some concerns about risk of bias.
Outpatient clinic visits for asthma exacerbation	No significant effect of wildfire smoke on asthma clinic visits with only one of three studies showing a positive association	9977 (3) [15,33,35]	Very low All observational studies, some concerns about risk of bias.
ED visits for any respiratory cause	Five of eight studies noted a positive association between wildfire smoke exposure and respiratory ED visits; two showed no difference and one showed a negative association	557,454 (8) [23,33,34,36,38,39,40,42]	Moderate Observational studies however participant numbers are high and some evidence of dose–response relationship
ED visits for asthma exacerbation	No significant association between wildfire exposure and ED asthma visits with three of eight studies showing a positive association and five no association	557,454 (8) [23,33,34,36,38,39,40,42]	Moderate Observational studies however participant numbers are high and some evidence of dose–response relationship
Hospitalization for any respiratory cause	Three of four studies showed a positive association between wildfire smoke and respiratory hospitalizations and one no association	13,258 (4) [30,31,33,38]	Moderate Some evidence of dose response
Hospitalization for asthma exacerbation	No significant association between asthma hospitalizations and wildfire smoke, with two of four studies showing a positive association and two no association	13,258 (4) [30,31,33,38]	Moderate Some evidence of dose response
Any respiratory symptoms or self-reported diagnoses: dry or wet cough, asthma exacerbation, bronchitis	No clear association between wildfire smoke and respiratory symptoms shown with one study positive and one smaller study showing no association	1330 (2) [15,16]	Very low Risk of bias with exposure characterization and outcome assessment
Itchy/watery eyes, nasal congestion or sneezing, rhinitis and sore throat	Strong association between eye, nose and throat symptoms and wildfire smoke exposure in two studies	1330 (2) [15,16]	Low Risk of bias for exposure characterization

^a^ A pooled estimate was not available for any of the outcomes due to the significant heterogeneity across studies. Instead, a qualitative synthesis of the evidence is reported.

## Data Availability

No new data were created or analyzed in this study. Data sharing is not applicable to this article.

## Supplementary Materials

The following are available online at https://www.mdpi.com/article/10.3390/ijerph18168799/s1, Detailed search strategy for systematic review.

## Appendix A

**Table A1:** PRISMA 2020 Checklist for Systematic Reviews [20].

Section and Topic	Item #	Checklist Item	Location Where Item is Reported
TITLE			
Title	1	Identify the report as a systematic review.	Title Page 1
ABSTRACT			
Abstract	2	See the PRISMA 2020 for Abstracts checklist.	Abstract Page 1
INTRODUCTION			
Rationale	3	Describe the rationale for the review in the context of existing knowledge.	Section 1 Page 2, line 28
Objectives	4	Provide an explicit statement of the objective(s) or question(s) the review addresses.	Section 1 Page 2, line 34
METHODS			
Eligibility criteria	5	Specify the inclusion and exclusion criteria for the review and how studies were grouped for the syntheses.	Eligibility: Section 2.3 Page 3 line 66 Grouping: Section 2.3 Page 3, line 84
Information sources	6	Specify all databases, registers, websites, organisations, reference lists and other sources searched or consulted to identify studies. Specify the date when each source was last searched or consulted.	Section 2.2 Page 2, line 49
Search strategy	7	Present the full search strategies for all databases, registers and websites, including any filters and limits used.	Section 2.2 Page 2, line 48; Supplementary Materials S1 Page 26, line 423
Selection process	8	Specify the methods used to decide whether a study met the inclusion criteria of the review, including how many reviewers screened each record and each report retrieved, whether they worked independently, and if applicable, details of automation tools used in the process.	Section 2.4 Page 3, line 93
Data collection process	9	Specify the methods used to collect data from reports, including how many reviewers collected data from each report, whether they worked independently, any processes for obtaining or confirming data from study investigators, and if applicable, details of automation tools used in the process.	Section 2.5 Page 3, line 100
Data items	10a	List and define all outcomes for which data were sought. Specify whether all results that were compatible with each outcome domain in each study were sought (e.g., for all measures, time points, analyses), and if not, the methods used to decide which results to collect.	Section 2.5 Page 3, line 103
	10b	List and define all other variables for which data were sought (e.g., participant and intervention characteristics, funding sources). Describe any assumptions made about any missing or unclear information.	Section 2.5 Page 3, line 103
Study risk of bias assessment	11	Specify the methods used to assess risk of bias in the included studies, including details of the tool(s) used, how many reviewers assessed each study and whether they worked independently, and if applicable, details of automation tools used in the process.	Section 2.6 Page 4, line 121
Effect measures	12	Specify for each outcome the effect measure(s) (e.g., risk ratio, mean difference) used in the synthesis or presentation of results.	Section 2.5 Page 3, line 110
Synthesis methods	13a	Describe the processes used to decide which studies were eligible for each synthesis (e.g., tabulating the study intervention characteristics and comparing against the planned groups for each synthesis (item #5)).	Section 2.7 Page 4, line 136
	13b	Describe any methods required to prepare the data for presentation or synthesis, such as handling of missing summary statistics, or data conversions.	Section 2.7 Page 4, line 137
	13c	Describe any methods used to tabulate or visually display results of individual studies and syntheses.	Section 2.7 Page 4, lines 137 and 149
	13d	Describe any methods used to synthesize results and provide a rationale for the choice(s). If meta-analysis was performed, describe the model(s), method(s) to identify the presence and extent of statistical heterogeneity, and software package(s) used.	Section 2.7 Page 4, line 135
	13e	Describe any methods used to explore possible causes of heterogeneity among study results (e.g., subgroup analysis, meta-regression).	NA
	13f	Describe any sensitivity analyses conducted to assess robustness of the synthesized results.	Section 2.7 Page 4, line 142
Reporting bias assessment	14	Describe any methods used to assess risk of bias due to missing results in a synthesis (arising from reporting biases).	Section 2.6 Page 4, line 123
Certainty assessment	15	Describe any methods used to assess certainty (or confidence) in the body of evidence for an outcome.	Section 2.7 Page 4, line 142
RESULTS			
Study selection	16a	Describe the results of the search and selection process, from the number of records identified in the search to the number of studies included in the review, ideally using a flow diagram.	Section 3.1 Page 4, line 154 and Figure 1
	16b	Cite studies that might appear to meet the inclusion criteria, but which were excluded, and explain why they were excluded.	Section 3.1 Page 4, line 163
Study characteristics	17	Cite each included study and present its characteristics.	Section 3.2 Page 19, line 176 and Table 1
Risk of bias in studies	18	Present assessments of risk of bias for each included study.	Section 3.7 Page 22, line 252
Results of individual studies	19	For all outcomes, present, for each study: (a) summary statistics for each group (where appropriate) and (b) an effect estimate and its precision (e.g., confidence/credible interval), ideally using structured tables or plots.	Section 3.2 Page 19, line 176 and Table 1
Results of syntheses	20a	For each synthesis, briefly summarise the characteristics and risk of bias among contributing studies.	Section 3.6–3.10 Page 21, line 241 and Table 2
	20b	Present results of all statistical syntheses conducted. If meta-analysis was done, present for each the summary estimate and its precision (e.g., confidence/credible interval) and measures of statistical heterogeneity. If comparing groups, describe the direction of the effect.	NA
	20c	Present results of all investigations of possible causes of heterogeneity among study results.	NA
	20d	Present results of all sensitivity analyses conducted to assess the robustness of the synthesized results.	NA
Reporting biases	21	Present assessments of risk of bias due to missing results (arising from reporting biases) for each synthesis assessed.	Section 3.7 Page 22, line 251 and Figure 2
Certainty of evidence	22	Present assessments of certainty (or confidence) in the body of evidence for each outcome assessed.	Section 3.8 Page 22, line 268 and Table 3
DISCUSSION			
Discussion	23a	Provide a general interpretation of the results in the context of other evidence.	Section 4.1 Page 24, line 309
	23b	Discuss any limitations of the evidence included in the review.	Section 4.2 Page 25, line 379
	23c	Discuss any limitations of the review processes used.	Section 4.3 Page 25, line 390
	23d	Discuss implications of the results for practice, policy, and future research.	Section 5 line 416
OTHER INFORMATION			
Registration and protocol	24a	Provide registration information for the review, including register name and registration number, or state that the review was not registered.	Section 2.1 Page 2, line 46
	24b	Indicate where the review protocol can be accessed, or state that a protocol was not prepared.	Section 2.1 Page 2, line 46
	24c	Describe and explain any amendments to information provided at registration or in the protocol.	Section 2.3 Page 3, lines 64 and 67
Support	25	Describe sources of financial or non-financial support for the review, and the role of the funders or sponsors in the review.	Funding: Page 26, line 431
Competing interests	26	Declare any competing interests of review authors.	Conflicts of Interest: Page 26, line 436
Availability of data, code and other materials	27	Report which of the following are publicly available and where they can be found: template data collection forms; data extracted from included studies; data used for all analyses; analytic code; any other materials used in the review.	Supplemental Materials

## Appendix B

**Table A2:** Systematic Review Without Meta-analysis (SWIM) checklist [24].

SWiM Reporting Item	Item Description	Page in Manuscript Where Item Is Reported
Grouping studies for synthesis	(1a) Provide a description of, and rationale for, the groups used in the synthesis (e.g., groupings of populations, interventions, outcomes, study design)	Section 2.7 Page 4, line 134
	(1b) Detail and provide rationale for any changes made subsequent to the protocol in the groups used in the synthesis	Section 2.3 Page 3, lines 64 and 67
2. Describe the standardised metric and transformation methods used	Describe the standardised metric for each outcome. Explain why the metric(s) was chosen, and describe any methods used to transform the intervention effects, as reported in the study, to the standardised metric, citing any methodological guidance consulted	Section 2.3 Page 3, line 68
3. Describe the synthesis methods	Describe and justify the methods used to synthesise the effects for each outcome when it was not possible to undertake a meta-analysis of effect estimates	Section 2.7 Page 3, line 134
4. Criteria used to prioritise results for summary and synthesis	Where applicable, provide the criteria used, with supporting justification, to select the particular studies, or a particular study, for the main synthesis or to draw conclusions from the synthesis (e.g., based on study design, risk of bias assessments, directness in relation to the review question)	Section 2.7 Page 3, line 142
5. Investigation of heterogeneity in reported effects	State the method(s) used to examine heterogeneity in reported effects when it was not possible to undertake a meta-analysis of effect estimates and its extensions to investigate heterogeneity	Section 2.7 Page 3, line 137
6. Certainty of evidence	Describe the methods used to assess certainty of the synthesis findings	Section 2.7 Page 3, line 142
7. Data presentation methods	Describe the graphical and tabular methods used to present the effects (e.g., tables, forest plots, harvest plots). Specify key study characteristics (e.g., study design, risk of bias) used to order the studies, in the text and any tables or graphs, clearly referencing the studies included	Section 2.6, Section 2.7 Page 3; Section 2.3 Page 2, Table 2 Page 21, Figure 2 Page 22
8. Reporting results	For each comparison and outcome, provide a description of the synthesised findings, and the certainty of the findings. Describe the result in language that is consistent with the question the synthesis addresses, and indicate which studies contribute to the synthesis	Table 3 Page 23, Sections 3.8–3.10 Page 22
Discussion		
9. Limitations of the synthesis	Report the limitations of the synthesis methods used and/or the groupings used in the synthesis, and how these affect the conclusions that can be drawn in relation to the original review question	Section 4.2 and Section 4.3 Page 25
