# Supplementary material for: Assessing the Risk of Respiratory-Related Healthcare Visits Associated with Wildfire Smoke Exposure in Children 0–18 Years Old: A Systematic Review
Source: Int J Environ Res Public Health. 2021 Aug 20;18(16):8799. doi: 10.3390/ijerph18168799 (PMC8392577; doi:10.3390/ijerph18168799)
Supplement: Supplementary file 1 [file ijerph-18-08799-s001.zip › ijerph-1297261-supplementary.pdf]

Supplementary Materials

OVID account: Wildfire/wildfire

Search strategy:

https://guides.library.ualberta.ca/c.php?g=342568&p=5096194

Search results

Database searches total number = 228 + 308 + 363 + 241 + 567 + 119 + 59 + 153 + 100 = 2138

After duplicate removal = 1165

Ovid MEDLINE(R) ALL 1946 to 18 December 2020

Date searched: 21 December 2020

Results: 228

1. (wildfire* or forestfire* or bushfire* or ((wild* or bush or forest* or vegetation or landscape) adj6 fire*)).mp.
2. exp child/or exp infant/or adolescent/or exp pediatrics/or minors/or (pediatric* or paediatric* or child* or newborn* or infan* or baby or babies or neonat* or preschool* or kindergarten* or kindergarden* or school* or ((day care* or daycare) not adult*) or toddler* or boy or boys or girl* or pubescen* or juvenile* or teen* or youth* or (student* not (undergraduate student* or graduate student* or college student* or university student*)) or adolesc* or pre-pubesc* or prepubesc*).mp. or (child* or adolesc* or pediat* or paediat*).jw.
3. 1 and 2

Embase 1974 to 2020 December 18 (OVID Interface)

Date searched: 21 December 2020

Results: 308

1. (wildfire* or forestfire* or bushfire* or ((wild* or bush or forest* or vegetation orlandscape) adj6 fire*)).mp.
2. exp child/or exp adolescent/or exp pediatrics/or juveniles/or (pediatric* or paediatric* or child* or newborn* or infan* or baby or babies or neonat* or preschool* or kindergarten* or kindergarden* or school* or ((day care* or daycare) not adult*) or toddler* or boy or boys or girl* or pubescen* or juvenile* or teen* or youth* or (student* not (undergraduate student* or graduate student* or college student* or university student*)) or adolesc* or pre-pubesc* or prepubesc*).mp. or (child* or adolesc* or pediat* or paediat*).jx.
3. 1 and 2

Web of Science (Indexes = SCI-EXPANDED, SSCI, A&HCI, CPCI-S, CPCI-SSH, BKCI-S, BKCI-SSH, ESCI, CCR-EXPANDED, IC)

Date searched: 21 December 2020

Results: 363

TS = (wildfire* OR forestfire* OR bushfire* OR ((wild* OR bush OR forest* OR vegetation OR landscape) NEAR/6 fire*)) AND (TS =

(pediatric* OR paediatric* OR child* OR newborn* OR infan* OR baby OR babies OR neonat* OR preschool* OR kindergarten* OR kindergarden* OR nursery-school* OR pre-school* OR elementary-school* OR schoolchild* OR high-school* OR secondary-school* OR junior-high OR ((day-care* OR daycare*) NOT adult*) OR toddler* OR boy OR boys OR girl* OR “pubescent” OR “pubescence” OR teen* OR youth* OR (student* NOT (undergraduate-student* OR grad*-student* OR college-student* OR university-student* OR engineering-student* OR comp*-sci*-student*)) OR adolesc* OR pre-pubesc* OR prepubesc*) OR SO = (child* OR adolesc* OR pediat* OR paediat*))

CABI: CAB Abstracts and Global Health (Clarivate Analytics interface)
Date searched: 21 December 2020
Results: 241
TS = (wildfire* OR forestfire* OR bushfire* OR ((wild* OR bush OR forest* OR vegetation OR landscape) NEAR/6 fire*)) AND (TS = (pediatric* OR paediatric* OR child* OR newborn* OR infan* OR baby OR babies OR neonat* OR preschool* OR kindergarten* OR kindergarden* OR nursery-school* OR pre-school* OR elementary-school* OR schoolchild* OR high-school* OR secondary-school* OR junior-high OR ((day-care* OR daycare*) NOT adult*) OR toddler* OR boy OR boys OR girl* OR “pubescent” OR “pubescence” OR teen* OR youth* OR (student* NOT (undergraduate-student* OR grad*-student* OR college-student* OR university-student* OR engineering-student* OR comp*-sci*-student*)) OR adolesc* OR pre-pubesc* OR prepubesc*) OR SO = (child* OR adolesc* OR pediat* OR paediat*))

Scopus
Date searched: 21 December 2020
Results: 567
TITLE-ABS-KEY (wildfire* OR forestfire* OR bushfire* OR ((wild* OR bush OR forest* OR vegetation OR landscape) W/6 fire*)) AND (TITLE-ABS-KEY (pediatric* OR paediatric* OR child* OR newborn* OR infan* OR baby OR babies OR neonat* OR preschool* OR kindergarten* OR kindergarden* OR nursery-school* OR pre-school* OR elementary-school* OR schoolchild* OR high-school* OR secondary-school* OR junior-high OR ((day-care* OR daycare*) AND NOT adult*) OR toddler* OR boy OR boys OR girl* OR {pubescent} OR {pubescence} OR teen* OR youth* OR (student* AND NOT (undergraduate-student* OR grad*-student* OR college-student* OR university-student* OR engineering-student* OR comp*-sci*-student*)) OR adolesc* OR pre-pubesc* OR prepubesc*) OR SRCTITLE (child* OR adolesc* OR pediat* OR paediat*))

CINAHL Plus with Full Text (EBSCOhost interface)
Date searched: 21 December 2020
Results: 119
(wildfire* or forestfire* or bushfire* or ((wild* or bush or forest* or vegetation or landscape) N6 fire*))
AND
((MH “Child+”) OR (MH “Adolescence+”) OR (MH “Minors (Legal)”) OR (pediatric* or paediatric* or child* or newborn* or infan* or baby or babies or neonat* or preschool* or kindergarten* or kindergarden* or school* or ((day care* or daycare*) not adult*) or toddler* or boy or boys or girl* or pubescen* or juvenile* or teen* or youth* or (student* not (undergraduate-student* or graduate-student* or college-student* or university-student*)) or adolesc* or pre-pubesc* or prepubesc*) or SO(child* or adolesc* or pediat* or paediat*))

Greenfile (EBSCOhost interface)
Date searched: 21 December 2020
Results: 59
S1 (TI(wildfire* or forestfire* or bushfire* or ((wild* or bush or forest* or vegetation or landscape) N6 fire*))) OR (AB((wildfire* or forestfire* or bushfire* or ((wild* or bush or forest* or vegetation or landscape) N6 fire*)))
S2 (TI(pediatric* or paediatric* or child* or newborn* or infan* or baby or babies or neonat* or preschool* or kindergarten* or kindergarden* or school* or ((day care* or daycare*) not adult*) or toddler* or boy or boys or girl* or pubescent or pubescence or teen* or youth* or (student* not (undergraduate-student* or graduate-student* or college-student* or university-student*)) or adolesc* or pre-pubesc* or prepubesc*)) OR (AB(pediatric* or paediatric* or child* or newborn* or infan* or baby or babies or neonat* or preschool* or kindergarten* or kindergarden* or school* or ((day care* or daycare*) not adult*) or toddler* or boy or boys or girl* or pubescent or pubescence or teen* or youth* or (student* not (undergraduate-student* or graduate-student* or college-student* or university-student*)) or adolesc* or pre-pubesc* or prepubesc*)) OR (SO(child* or adolesc* or pediat* or paediat*))
S3 S1 AND S2
Limit to academic journals

Proquest Earth, Atmospheric & Aquatic Science Database
Date searched: 21 December 2020
Results: 153
noft(wildfire* OR forestfire* OR bushfire* OR ((wild* OR bush OR forest* OR vegetation OR landscape) NEAR/6 fire*)) AND noft(pediatric* OR paediatric* OR child* OR newborn* OR infan* OR

baby OR babies OR neonat* OR preschool* OR kindergarten* OR kindergarden* OR nursery-school* OR pre-school* OR elementary-school* OR schoolchild* OR high-school* OR secondary-school* OR junior-high OR ((day-care* OR daycare*) NOT adult*) OR toddler* OR boy OR boys OR girl* OR "pubescent" OR "pubescence" OR teen* OR youth* OR (student* NOT (undergraduate-student* OR grad*-student* OR college-student* OR university-student* OR engineering-student* OR comp*-sci*-student*)) OR adolesc* OR pre-pubesc* OR prepubesc*)
Limit to Scholarly Journals, books, conference papers, reports, Dissertations & Theses

HERO- Health and Environmental Research Online
https://hero.epa.gov/hero/index.cfm/search (21 December 2020)
Date searched: 21 December 2020
Results: Took top 100 results only (June 2020) Update searching 23 December 2020 (search results limited to 2020) yielded 0 studies
Search For: “children” (match all words)
Search For: wild-fire wildfire forest-fire forestfire vegetation-fire bush-fire landscape-fire (match any word)
Sort by search score highest to lowest

Table S1. Detailed breakdown of risk of bias analysis. DL = definitely low risk of bias; PL = probably low risk of bias; PH = probably high risk of bias; DH = definitely high risk of bias. Each reviewer's score is shown separately.

	Comparison Groups	Confounding and Modifying Variables	Outcome Data Complete	Exposure Characterization	Outcome Assessment	All Measured Outcomes Reported	Other Validity Issues	Decision
Delfino et al. [30]	PL	PL	PL	PL	PL	PL	No	
	PL	DL	PL	PL	PL	PL	No	Include
Gan et al. [31]	PL	PL	PL	PL	PL	PL	No	
	PL	PL	PL	PL	PL	PL	No	Include
Hanigan et al. [32]	PL	PH	PH	PH	PL	PL	No	
	PL	PH	PH	PH	PL	PL	No	Include
Henderson et al. [23]	PL	PL	PL	PL	PL	PL	No	
	PL	PL	PL	PL	PL	PL	No	Include
Hutchinson et al. [33]	PL	PL	PL	PL	PL	PL	No	
	PL	PL	PL	PL	PL	PL	No	Include
Johnston et al. [34]	PL	PL	PL	PL	PL	PL	No	
	PL	PL	PL	PL	PL	PL	No	Include
Kunzli et al. [15]	PL	PL	PH	PL	PH	PL	No	
	PL	PL	PH	PL	PH	PL	No	Include
Lee et al. [35]	PL	PL	PH	PH	PL	PL	No	
	PL	PL	PH	PH	PL	PL	No	Include
Liebel et al. [36]	PL	PL	PL	PL	PH	PL	No	
	PL	PL	PL	PL	PH	PL	No	Include
Mott et al. [29]	PH	PH	PL	PH	PL	PL	No	
	PL	PH	PL	DH	PL	PL	Yes *	Exclude
Pratt et al. [37]	PL	PL	PL	PH	PL	PL	No	
	PL	PL	PL	PH	PL	PL	No	Include
Reid et al. [38]	PL	DL	DL	DL	PL	PL	No	
	PL	DL	PL	DL	PL	PL	No	Include
Resnick et al. [39]	PL	PH	PL	PL	PL	PL	No	
	PL	PH	PL	PL	PL	PL	No	Include
Stowell et al. [40]	PL	DL	PL	NA	NA	PL	No	
	PL	DL	DL	PL	PL	PL	No	Include
Tham et al. [41]	NA	PL	PL	PL	PL	PL	No	
	PL	PL	PL	PL	PL	PL	No	Include
Tinling et al. [42]	PL	PL	PL	PL	PL	PL	No	
	PL	PL	PL	PL	PL	PL	No	Include
Vicedo et al. [16]	PL	PL	PL	PH	PH	PL	No	
	PL	PL	PL	PH	PH	PL	No	Include

* Statistical methods inappropriate, did not account for confounding and modifying variables.
